# On the Origin and Prevention of Some of the Diseases of Human Teeth and Bones

**Published:** 1799-11

**Authors:** 

**Affiliations:** New-York


### *On the Origin and Prevention of ſome of the Diſeaſes of Human Teeth and Bones. By Dr.* MITCHILL, *of New-York.*

IN the ſeventh Number of this Journal, under the ſection containing "Medical and Phyſical Intelligence," p. 183 and ſeq., we have already communicated to our readers the introductory part of an ingenious paper, "*On the Application of the Doctrine of the Septic Fluids, to explain ſome of the Diſeaſes of Human Teeth and Bones.*" As the ſubſequent part of this inquiry is more immediately connected with medical practice than that formerly inſerted, we think it entitled to a place in this department of our Journal.

Phoſphoric acid prefers lime to alkalis, and therefore alkalis united with it are immediately rendered turbid by lime-water; and a ſaline powder, very difficultly ſoluble in water is depoſited, conſiſting of lime ſaturated with phoſphoric acid. Alkalis, therefore, whether fixed or volatile, would ſeem to be incapable of deſtroying the ſolid matter of the teeth, whatever their action may be upon the gums. Lime may be diſengaged from its connexion with phoſphoric acid by the oxalic, ſulphuric, ſeptic (nitric), and tartaric acids. Conſequently, acid of ſugar, ſpirit of vitriol, aquafortis, and cream of tartar, may decompoſe the teeth, by attracting the lime, and diſengaging the phoſphoric acid. The ſeptic acid, after barytes, pot-aſh, and ſoda, has the next ſtrongeſt attraction for lime, and after this for magneſia, ammoniac, and clay.

Thus ſeptic acid, if formed from the remains of animal and vegetable ſubſtances, lurking about or among the teeth, in attaching itſelf to the lime, will detach the acid of phoſphorus. This, added to the matters already emitting their ſcents, will have a tendency to increaſe the offenſiveneſs of the breath.

Whatever contributes to the accumulation of the matters from which ſeptic acid is produced, may be expected to injure the teeth. Hence lying-in-women, and perſons ſuffering long fits of ſickneſs, are particularly expoſed to the cauſes which deſtroy them; and this the more rapidly, becauſe, in ſuch ſituations, it often happens that little or no aſſiſtance is afforded by art, in removing thoſe things which, by their preſence and accumulation, occaſion the miſchief. When formed in the mouth, it may mingle likewiſe with the ſpittle, and vitiate the *guſtatory powers of the tongue and palate*, and, when ſwallowed, may impede the healthy *functions of the ſtomach*. Hence may be explained one ſpecies of anorexia, anorexia, especially that mentioned by DARWIN (2. Zoonomia, class ii. ord. 2. gen. 2. sp. 1.), where "want of appetite is sometimes produced by the putrid matter from many decaying teeth, being perpetually mixed with the saliva, and thence affecting the organ of taste, and greatly injuring the digestion." The formation of such a substance in the mouth enters deeply into the explanation of the symptoms of fevers, particularly the condition of the teeth, gums, tongue, and throat; with vitiated taste, thirst, aphthæ, colour of the tongue, &c.

If an incrustation containing septic matter is thus formed, and is a calcareous composition, of a kind different from the teeth, it is possible to remove it by chemical agents, which have not the power of decomposing the teeth; for as barytes, pot-ash, and soda, can disengage the *septic acid* from the lime, so either of these substances may be serviceable in removing the concretion, and, at the same time, not endangering the teeth, whose *phosphoric acid* having a greater attraction for lime than for alkalies, is incapable of being displaced by them. Alkaline dentifrices would, therefore, appear capable of removing the calcareo-septic incrustations from the teeth, but incapable of corroding the teeth themselves. DEBOZE observes, that tobacco ashes (*La Pratique de Medecine de Laz. Reviere*, &c. L. vi. ch. 2) possess a surprising (tres-merveilleuse) power to cleanse and whiten the teeth. The active ingredient must be the pot-ash. The practice of some ladies of New-York confirms this.

As the septite of lime, however, is very deliquescent, there is, perhaps, only a moderate portion of the septic acid contained in the stony tartar of the teeth. There is another form in which it is peculiarly destructive. Dentists distinguish tartar into three species, viz. the *yellow*, the *black*, and the *green*: of these, the last is observed to be by far the most pernicious; it never forms a crust or petrifaction, but always appears like a *green stain*. The enamel of the teeth beneath it is generally corroded, and almost always eaten through, or destroyed. This is, doubtless, owing to the *septic acid* formed under the edge of the gum, or between the teeth, from the remains of food containing septon, which, aided by the heat and moisture of the mouth, affords that poisonous and destructive fluid, by uniting with oxygen. Dr. MITCHILL believes, that a small portion of this acid, formed thus in the mouth, and adhering to a sound tooth, is the cause of that violent and sometimes fatal disease, consequent upon transplanting these bony substances into the bleeding jaws of another person. The cases are seldom or never venereal.

But

But the feptic acid formed in thefe inftances, by corruption on the furface of a tooth, poifons the patient of the dentift when inferted into the frefh focket, in the fame manner that it poifons diffectors when their wounded fingers receive it from the furface of a putrefying mufcle.

The fafe method of preparing teeth for tranfplanting, is to wafh them repeatedly, *before* extraction, with a weak folution of carbonat of pot-afh, in water, to remove the feptic acid and other foul matters. This is, doubtlefs, preferable to wafhing the tooth in alkaline ley, *after* it has been drawn, as thereby its capability to grow faft would be endangered.

The feptic acid being thus capable of corroding the teeth and the alveolar proceffes of the jaws, who fhall affirm that its operation ftops there? Is it not taken in with our air, food, and drink? Are there not inftances of nodes and excreffences of bones, that are not wholly unlike the incruftations of the teeth? And are there not likewife inftances of caries of the offeous parts, which have a near fimilitude to the rottennefs of the inftruments employed in chewing our food?

In addition to the difeafes of the teeth and their fockets, from feptic acid produced in the mouth, as already ftated, and partly upon the interpretation of the facts of Mr. HUNTER; it would be very eafy to quote many authorities from books. Inftead, however, of difplaying much reading, Prof. MITCHILL contents himfelf with referring to the authority of an intelligent and fkilful furgeon, Mr. Ruffell, who publifhed, in 1794, a Practical Effay on Necrofis, wherein a bone or part of a bone dies, and a new one is re-produced to fupply its place. The *lower jaw-bone* is frequently difordered in this manner, (p. 87); its death and feparation often take place from *difeafe in the teeth and gums*; which, from their fituation, naturally determine the complaint to begin at the upper part, and proceed downwards, (p. 80); and cafes of necrofis of the lower jaw may be traced to be the effects of blows, and of *tooth-ach*, efpecially if a violent attack of inflammation has been excited by the application of any *acrid fubftance* to a carious tooth, &c. (p. 98); and feldom happen to perfons under 30 years of age, (p. 93).

It would feem, therefore, that the reafoning is fair and fafe, to confider this caries of maxillary bone as of a nature quite fimilar to the decompofition of the teeth and difordered condition of their fockets, which has been fhown to be connected with the formation of feptic acid in the mouth, and its corroding effect there.

[To be concluded in our next.]

